# A special issue preface: Radiocarbon in the Anthropocene

**DOI:** 10.1098/rsta.2022.0209

**Published:** 2023-11-27

**Authors:** Timothy I. Eglinton, Heather D. Graven, Peter A. Raymond, Susan E. Trumbore

**Affiliations:** ^1^ Department of Earth Sciences, ETH Zurich, Zurich 8092, Switzerland; ^2^ Department of Physics, Imperial College London, London, UK; ^3^ School of the Environment, Yale University, New Haven, CT, USA; ^4^ Department of Biogeochemical Processes, Max Planck Institute for Biogeochemistry, Jena 07745, Germany

**Keywords:** radiocarbon (^14^C), carbon cycle, climate change, Anthropocene, fossil fuels, bomb ^14^C

## Abstract

The Anthropocene is defined by marked acceleration in human-induced perturbations to the Earth system. Anthropogenic emissions of CO_2_ and other greenhouse gases to the atmosphere and attendant changes to the global carbon cycle are among the most profound and pervasive of these perturbations. Determining the magnitude, nature and pace of these carbon cycle changes is crucial for understanding the future climate that ecosystems and humanity will experience and need to respond to. This special issue illustrates the value of radiocarbon as a tool to shed important light on the nature, magnitude and pace of carbon cycle change.

This article is part of the Theo Murphy meeting issue 'Radiocarbon in the Anthropocene'.

## 1. Introduction

Radiocarbon, the radioactive isotope of carbon, serves as a powerful tool to examine carbon cycle processes over a range of timescales by virtue of two phenomena: (i) its quasi-constant production in the atmosphere and decay with an approximately 5700-year half-life and (ii) the abrupt spike of ‘bomb’ ^14^C introduced into the atmosphere in the mid twentieth century as a consequence of the initiation and cessation of above-ground nuclear weapons testing. Together, these phenomena allow tracking, apportionment and assessment of carbon exchange and turnover times between and within Earth's carbon reservoirs. Presently, the radiocarbon content of the atmosphere is returning to pre-industrial levels as a consequence of the redistribution of bomb ^14^C between atmospheric, oceanic and terrestrial reservoirs, as well as on-going emissions of ^14^C-depleted carbon from fossil fuel burning and land-use change. Future variations of ^14^C within and between different carbon reservoirs will depend on how trajectories of Anthropocene change evolve in the context of greenhouse gas emission scenarios and the efficacy of carbon reduction strategies.

With a backdrop of on-going environmental change, together with an appreciation of the unique value of ^14^C as a tool to understand the changing carbon cycle, a Royal Society-sponsored meeting on *Radiocarbon in the Anthropocene* was convened on 16 and 17 May 2022 at Whittlebury Park, UK, in which carbon cycle scientists and radiocarbon experts discussed current research related to the application of ^14^C to understand natural carbon cycle processes and to assess past and future changes. The theme issue includes papers stemming from presentations at this meeting, augmented by others that reflect current frontiers in radiocarbon research.

## 2. Contents of the theme issue

The first paper of the theme issue, an Opinion Piece by Eglinton *et al.* [1], seeks to highlight the value of ^14^C for understanding Anthropocene carbon cycle change, and to propose an intense phase of coordinated and sustained research activity that harnesses the full potential of radiocarbon.

Three papers describe applications of radiocarbon to assess soil carbon dynamics, and the factors controlling soil carbon turnover. The paper by Stoner *et al.* [2] describes the application of serial or ramped thermal oxidation of subsoils developed on different bedrock lithologies in order to examine the influence of mineralogy on organic matter stabilization and carbon quality. The products released were examined for molecular and isotopic (radiocarbon) composition and in the context of mineral composition, particularly pedogenic oxides and clays. Their findings provide strong evidence for mineralogical control on the age distribution, chemical diversity and persistence of soil organic matter, with implications for modelling soil C turnover, and for predictions of soil carbon vulnerability in the face of changing environmental conditions. Sierra *et al.* [3] present a modelling study that seeks to describe the transit time of carbon through the terrestrial biosphere, from photosynthetic carbon fixation to respiration. Variables that are explored include the impact of increasing productivity and respiration. Model results indicate that temperature-driven increases in respiration are fuelled by the degradation of older carbon pools, resulting in an increase in transit time, whereas the opposite holds for increased primary productivity. Simulations using different terrestrial carbon models indicate overall shorter transit times prevail due to the dominance of temperature-driven increases in productivity over the twentieth century. However, increased asymmetry in transit time distributions reflects the differing carbon dynamics of low- versus high-latitude ecosystems, with decreasing transit times in the former and increasing transit times in the latter due to the respiration of long-stored carbon. In an observational study that relates to the findings of Sierra *et al.*, Schuur *et al.* [4] describe results from almost 20 years of time-series CO_2_ flux and radiocarbon measurements at a permafrost site in Alaska where carbon has been stored in frozen and waterlogged soils that shed light on long-term ecosystem and carbon cycle perturbations in a region of the world that is experiencing the accelerated climate change. The detection of respired CO_2_ with depleted ^14^C values relative to atmospheric CO_2_ provides evidence of old permafrost soil C degradation, and a steeper trajectory in respired ^14^CO_2_ decrease relative to the atmosphere over the time series indicates that this degradation is enhanced with regional climate change, with temperature and moisture being the key variables. These findings suggest that changing environmental conditions are favouring release of old, previously stabilized carbon, representing a positive feedback with respect to on-going climate change.

Two manuscripts focus on the use of radiocarbon to examine controls on carbon dynamics during mobilization and transport along the aquatic continuum. Rhyner *et al.* [5] examined radiocarbon signatures of dissolved and particulate carbon phases in a suite of 21 Swiss rivers sampled during an interval of elevated water and sediment discharge that span a broad range of watershed characteristics. They found that mean river basin elevation was most strongly linked to ^14^C values, suggesting that the dynamics of carbon mobilized and exported by rivers is subject to significant ecoregional control. Gies *et al.* [6] use radiocarbon measurements of different groups of source-specific ‘biomarker’ compounds isolated from river sediments to examine relationships with watershed properties and carbon dynamics within river basins. They find coherence between ^14^C characteristics of terrestrial plant biomarkers and markers of soil microbial residues, suggesting that biospheric carbon export from watersheds is modulated by soil storage.

Two papers address ^14^C measurements of atmospheric CO_2_ in the context of constraining local to regional fossil fuel CO_2_ sources and emissions. Maier *et al*. [7] discuss the complexities in constraining fossil fuel CO_2_ surplus (ΔffCO_2_) emissions at a regional scale through the conventional approach of comparing local ^14^CO_2_ measurements to measurements at a background site. Specific challenges, biases and uncertainties are discussed in the context of the Integrated Carbon Observation System (ICOS) atmospheric network for Europe and the background station at Mace Head, Ireland. They highlight the significant biases that can be incurred in ΔffCO_2_ estimates as a consequence of the small signals as well as the impacts of nuclear contamination and heterotrophic respiration. Young *et al.* [8] describe the use of ^14^C as part of urban flask measurements from Auckland New Zealand that form part of a programme to assess local ffCO_2_ as well as excess carbon monoxide emissions relative to background. They determine that excess CO largely results from traffic emissions, and this information is used to develop a revised inventory for excess CO.

The final paper in the theme issue by Bard *et al.* [9] reports annual-resolution ^14^C measurements on tree rings from subfossil Scots Pines from the Southern French Alps in the context of past atmospheric ^14^C variability spanning a 700-year interval during the Late Glacial period. The record reveals two prominent events that are attributed to solar activity, providing further linkages with Greenland ice core records and temporal context for interpretation of past climate variability.

## Data Availability

This article has no additional data.
